# Supplementary material for: The kinase NEK6 positively regulates LSD1 activity and accumulation in local chromatin sub-compartments
Source: Commun Biol. 2024 Nov 10;7:1483. doi: 10.1038/s42003-024-07199-x (PMC11551153; doi:10.1038/s42003-024-07199-x)
Supplement: Supplementary file 2 — Supplementary Information [file 42003_2024_7199_MOESM2_ESM.pdf]

The kinase NEK6 positively regulates LSD1 activity and accumulation in local chromatin sub-compartments

Franziska Knodel[1], Jürgen Eirich[3], Sabine Pinter[1], Stephan A. Eisler[2], Iris Finkemeier[3], Philipp Rathert[1*]

[1] Department of Biochemistry, Institute of Biochemistry and Technical Biochemistry, University of Stuttgart, Stuttgart, Germany.

[2] Stuttgart Research Center Systems Biology (SRCSB), University of Stuttgart, Stuttgart, Germany.

[3] Institute of Plant Biology and Biotechnology, University of Münster, Münster, Germany

* To whom correspondence should be addressed. Tel: +49-711-685-64388; Fax: +49-711-685-64392; Email: philipp.rathert@ibtb.uni-stuttgart.de

Supplementary Figure 1: Knockdown validation of NEK6 shRNAs and cellular localization of LSD1 and NEK6 during different cell cycle phases.

Supplementary Figure 2: NEK6 phosphorylates different amino acids of LSD1 in vitro and S126 is the main phosphorylation target of NEK6.

Supplementary Figure 3: Condensation of the LSD1 IDR under various conditions in vitro is affected by S126ph.

Supplementary Figure 4: Condensation of LSD1 in living cells.

Supplementary Figure 5: Corelet system reveals necessity of FL-LSD1 and is guided by specific interaction of LSD1 and NEK6.

Supplementary Figure 6: Flow cytometry gating strategy.

Supplementary Figure 7: Uncropped pictures related to main figures.

Supplementary Figure 8: Uncropped pictures related to supplementary figures.

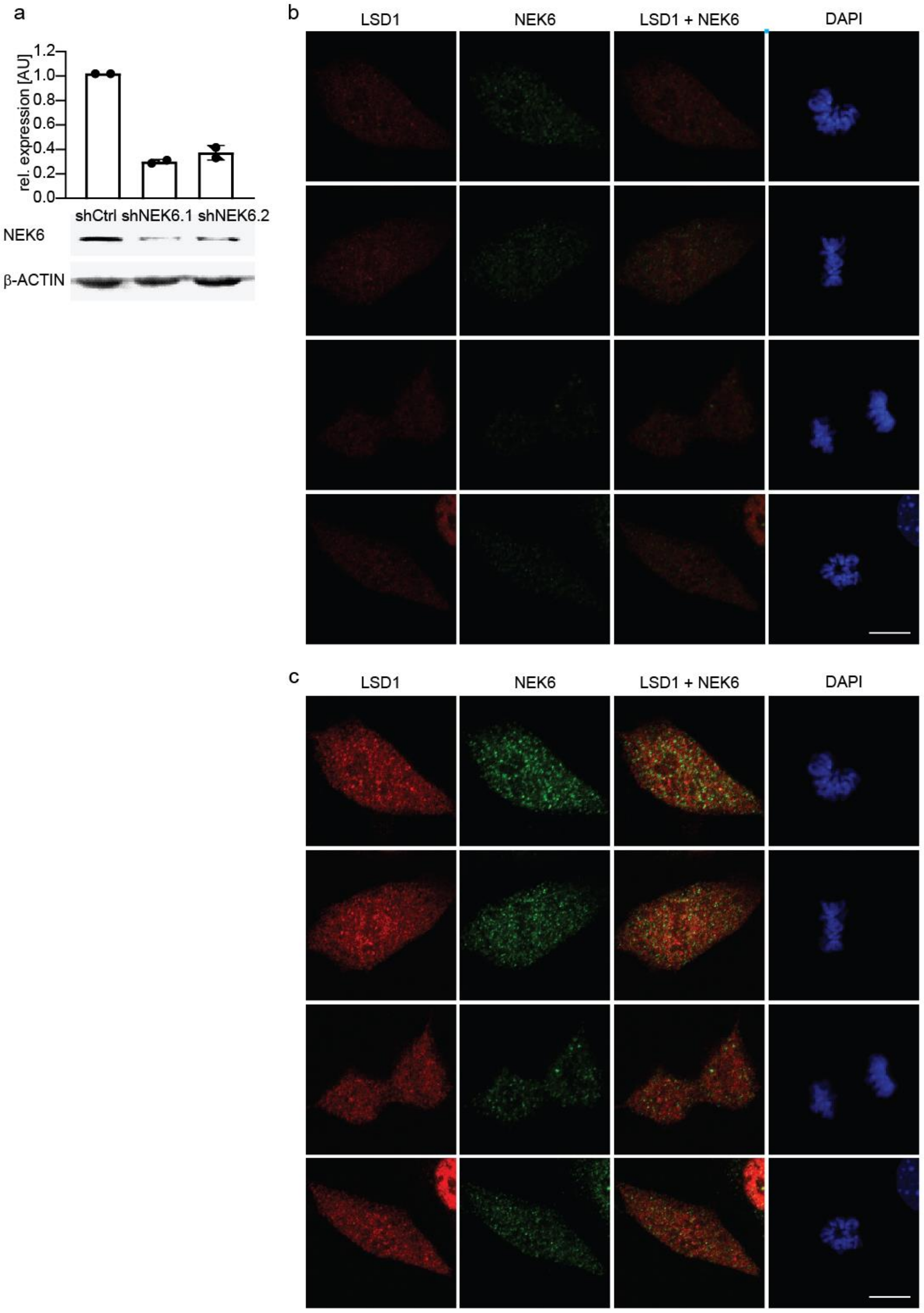

Supplementary Fig. 1: Knockdown validation of NEK6 shRNAs and cellular localization of LSD1 and NEK6 during different cell cycle phases.

a) Knockdown efficiency of applied shRNAs targeting NEK6 in NIH/3T3 reporter cells. 13 days after shRNA transduction, cells were harvested and protein lysate was

analysed via Western blot. Top: relative NEK6 expression normed to β-actin for n=2 independent transductions. Bottom: representative western blot image.

b) Representative immunofluorescence microscopy images of NIH/3T3 cells in different mitosis states stained for endogenous LSD1 and NEK6 show no colocalization. Scale bar = 10 µm

c) Same pictures as shown in b) with strongly increased signal intensity for better visualization.

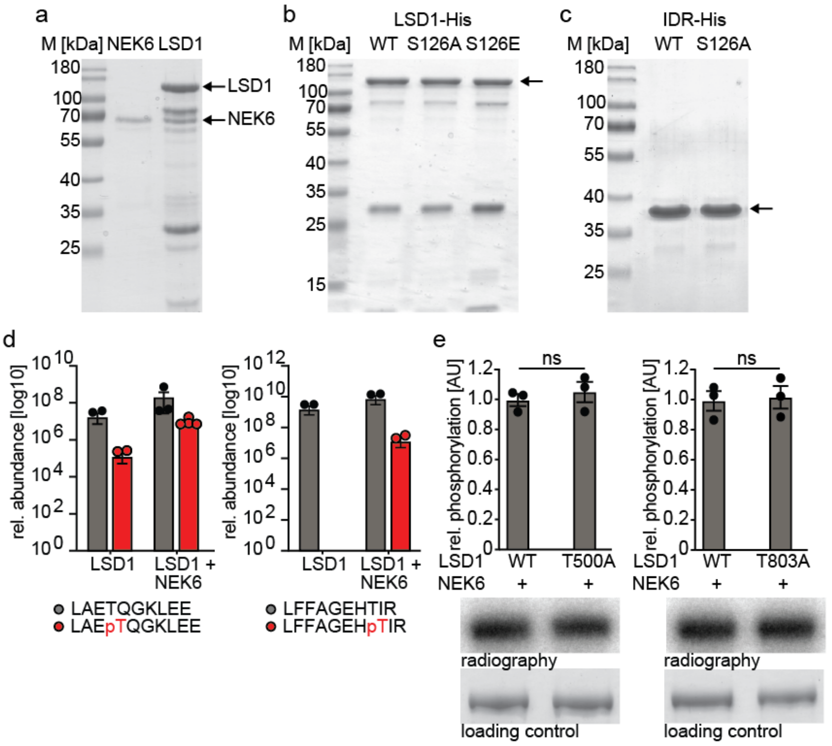

Supplementary Fig. 2: NEK6 phosphorylates different amino acids of LSD1 in vitro and S126 is the main phosphorylation target of NEK6.

a) Representative SDS-PAGE picture of recombinant GST-NEK6 and LSD1-His. The arrow marks the corresponding protein band.

b) Representative SDS-PAGE picture of LSD1-His WT, LSD1-His S126A and S126E. The arrow marks the corresponding protein band.

c) Representative SDS-PAGE picture of IDR-His WT and IDR-His S126A. The arrow marks the corresponding protein band.

d) LC-MS/MS intensity average of the detected peptide harbouring either T500 (left) or T803 (right) of LSD1.

e) Quantification of LSD1 T500A (left) or T803A (right) phosphorylation signal measured by autoradiography. Coomassie Brilliant Blue stained 12% SDS-polyacrylamide gel served as a loading control. (n=3, mean ± SEM, unpaired t-test, ns: not significant).

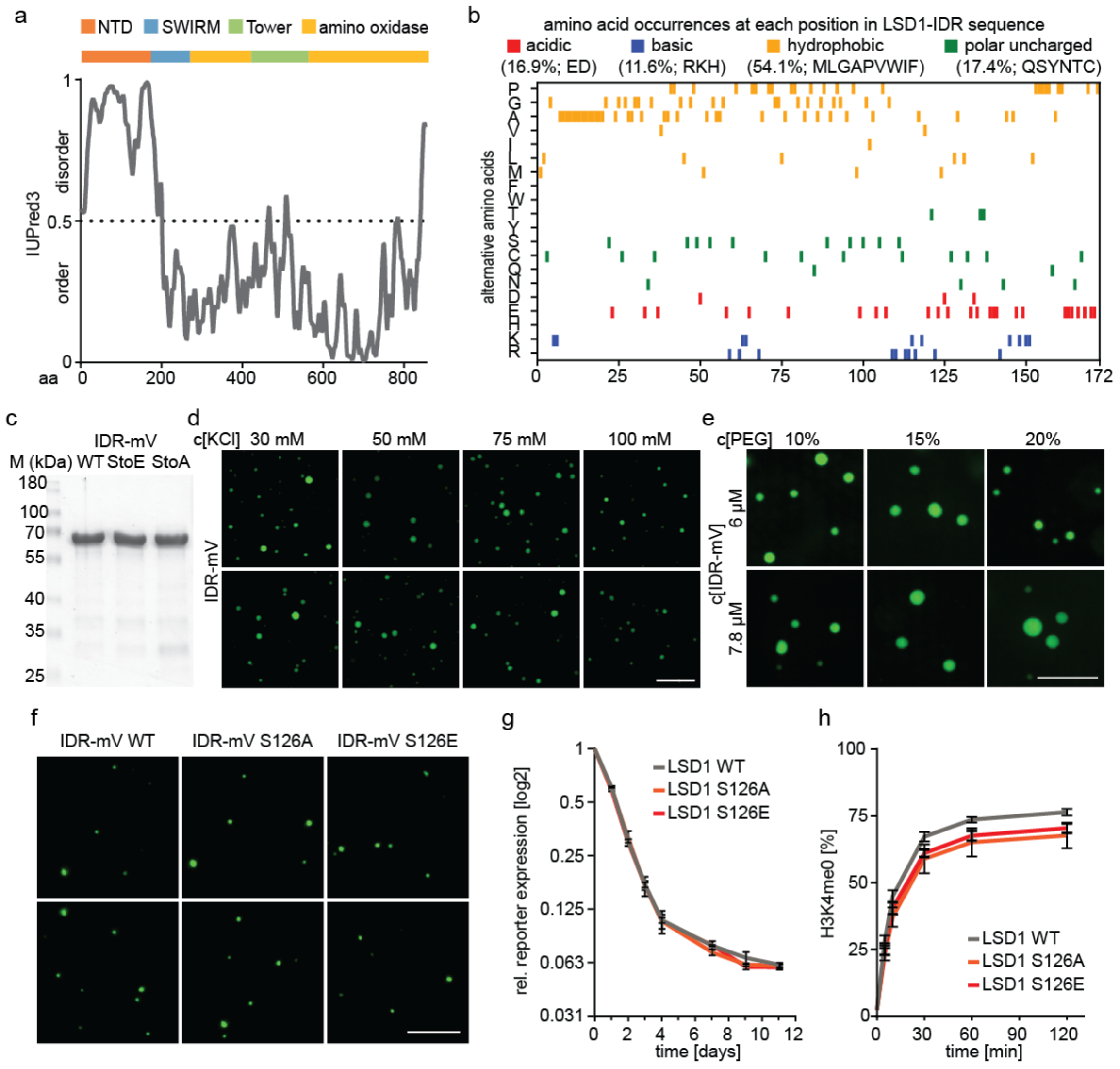

Supplementary Fig. 3: Condensation of the LSD1 IDR under various conditions in vitro is affected by S126ph.

a) Intrinsic order prediction of LSD1 using IUPred3 algorithm with x-axis depicting numbers of residues and y-axis showing degree of folding prediction.

b) Amino acid distribution in the LSD1-IDR divided in different categories (acidic, basic, polar uncharged and non-polar). Net charge at pH 7: -9.06; isoelectric point: 4.46.

c) Representative SDS-PAGE picture of recombinant IDR-mV-His WT and S126E or S126A mutant.

d) Representative immunofluorescence imagines depicting in vitro phase separated droplets of IDR-mV with increasing concentrations of KCl. Scale bar = 10 µm.

e) Representative immunofluorescence imagines depicting in vitro phase separated droplets of IDR-mV with increasing concentrations of PEG. Scale bar = 10 µm.

f) Representative immunofluorescence imagines depicting in vitro phase separated droplets of IDR-mV-WT vs. S126A and S126E. Scale bar = 10 µm.

g) mCherry reporter silencing was analysed in NIH/3T3 reporter cells when rTetR-LSD1 WT or S126 substituted with E or A is recruited over time. No difference was observed for respective LSD1 mutants. The median reporter expression of cells with Dox-induced rTetR-LSD1 recruitment was normalized to reporter cells without rTetR-LSD1 recruitment. (n=3 ± SEM)

h) In vitro demethylation activity of LSD1 WT or S126 substituted with E or A on monomethylated H3K4 peptide shows similar demethylation rates for all analysed LSD1 variants. (n=3 ± SEM)

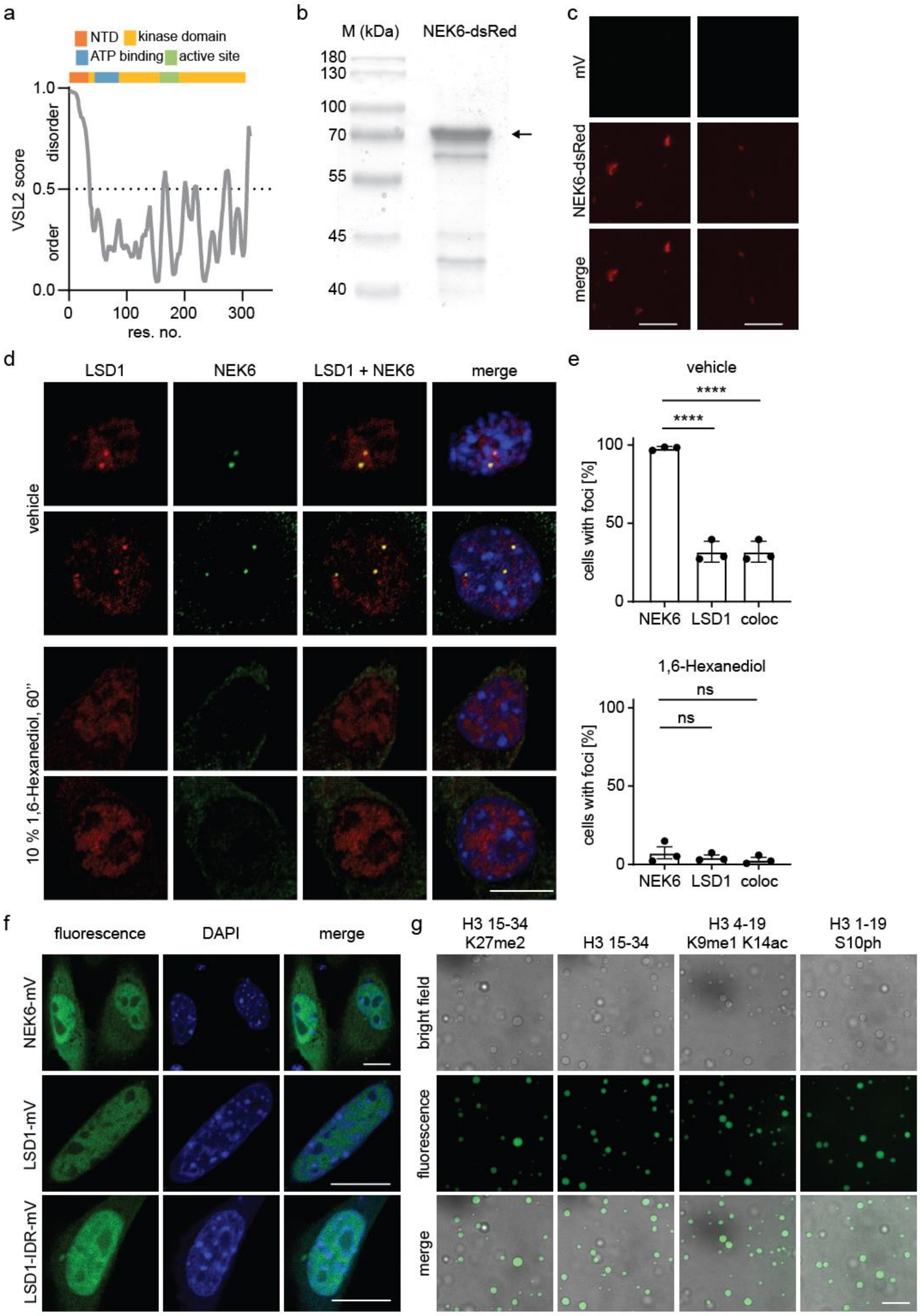

Supplementary Fig. 4: Condensation of LSD1 in living cells.

a) Intrinsic order prediction and domain structure overview of NEK6 by PONDR VSL2 algorithm. The X-axis depicts residue numbers plotted against the degree of folding prediction.

b) Representative SDS-PAGE image of recombinant NEK6-dsRed. Arrow marks corresponding protein band.
c) Representative fluorescence microscopy images depicting in vitro fibre-like structures of NEK6-dsRed. mVenus is not incorporated within these structures. Scale bar = 10 µm.
d) Representative immunofluorescence microscopy images of NIH/3T3 cells stained for LSD1 and NEK6 treated with vehicle or 10% hexanediol for 1 min. Prominent colocalized nuclear spots are vanished after treatment. Staining and treatment was performed in n=3 independent replicates with a total of N=587 cells for vehicle-treatment and N=293 cells for 1,6-Hexanediol treatment. Scale bar = 10 µm.
e) Bar graph showing the percentage of cells with prominent droplet-like localization of LSD1 and NEK6 within the nucleus together with colocalization of both proteins. (mean ± SEM, n=3 independent replicates with N=587 cells for vehicle-treatment and N=293 cells for 1,6-Hexanediol treatment, ordinary one-way ANOVA followed by Tukey's multiple comparison, ****:p<0.0001, ns: not significant)
f) Representative immunofluorescence images of NIH/3T3 cells ectopically expression NEK6-mV, the catalytically inactive K661A LSD1 mutant fused to mV (LSD1-mV) or the IDR-mV. DAPI serves as nuclear marker. Scale bar = 10 µm.
g) Representative fluorescence and bright filed microscopy images demonstrating incorporation of several histone H3.1 peptides labelled with FITC (peptide length as indicated) and harbouring different modifications into phase-separated droplets formed by IDR-His. Scale bar = 10 µm.

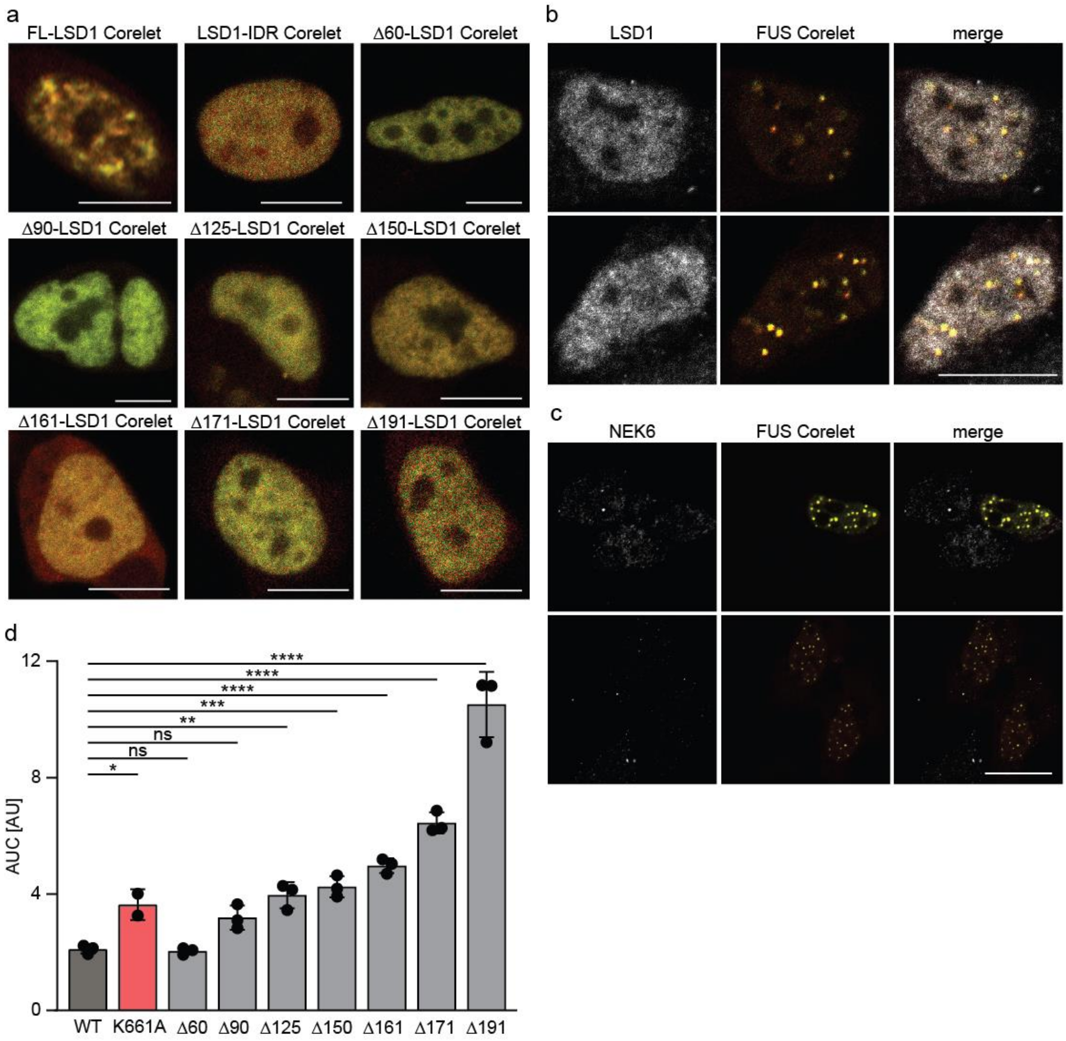

Supplementary Fig. 5: Corelet system reveals necessity of FL-LSD1 and is guided by specific interaction of LSD1 and NEK6.

a) Representative live cell fluorescence microscopy images of HEK293 cells expressing different catalytically inactive LSD1-K661A Corelet constructs during blue light activation. Only LSD1 FL is capable of Corelet droplet formation, whereas all N-terminally shortened LSD1-variants fail to condensate. Scale bar = 10 µm.

b) Statistical analysis of reporter silencing data in Fig. 5i was performed by a one-way ANOVA of the area under curve (AUC) followed by Dunnett´s multiple comparisons test. (n=3, mean ± SEM; ****: $p < 0.0001$, **: $p < 0.001$)

c) and d) Representative immunofluorescence images of HEK293 cells expressing FUS Corelet system with endogenous protein staining against LSD1 (c) or NEK6 (d). Corelet condensation was induced for 4 h with 5 min on/off cycles and proteins were fixed during blue light emission. Neither LSD1 nor NEK6 could be detected in FUS Corelet condensates. Scale bar = 10 µm.

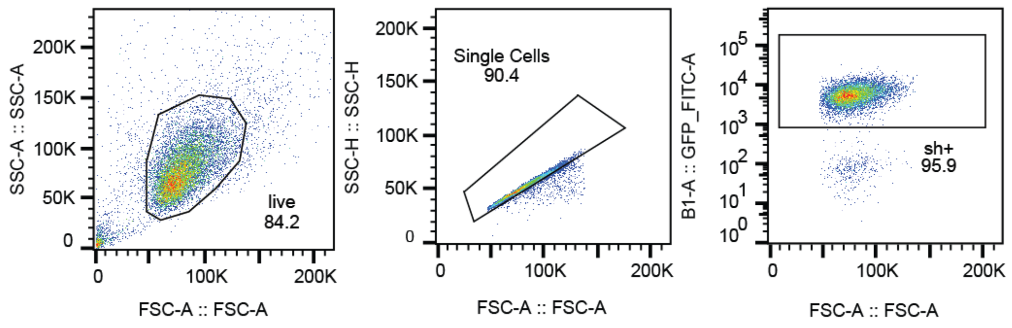

Supplementary Fig. 6: Flow cytometry gating strategy. All counts were analysed in scatterplots of Forward-Scatter-Area over Sideward-Scatter-Area to detect live cells (live). Single cells were determined in scatterplots of Forward-Scatter-Area over Forward-Scatter-Height (Single Cells). In case of reporter experiments including expression of shRNAs, their expression was evaluated in a scatterplot of Forward-Scatter-Area over GFP-FITC_Area (sh+).

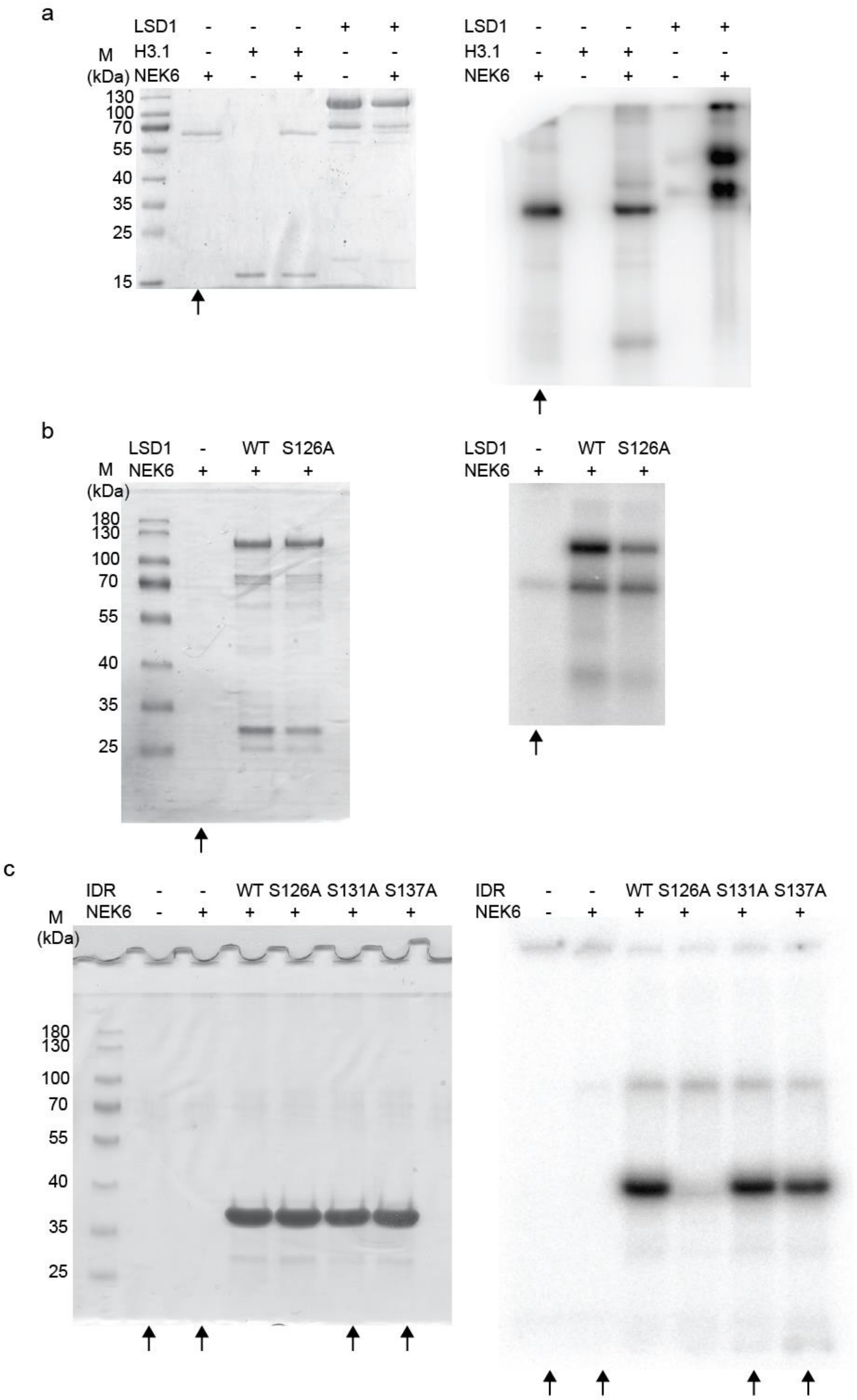

Supplementary Fig. 7: Uncropped SDS-PAGE pictures and corresponding radiography pictures related to a) Fig. 2a., b) Fig. 2d. and c) Fig. 2e. Arrows mark lanes that are not shown in the connected figures.

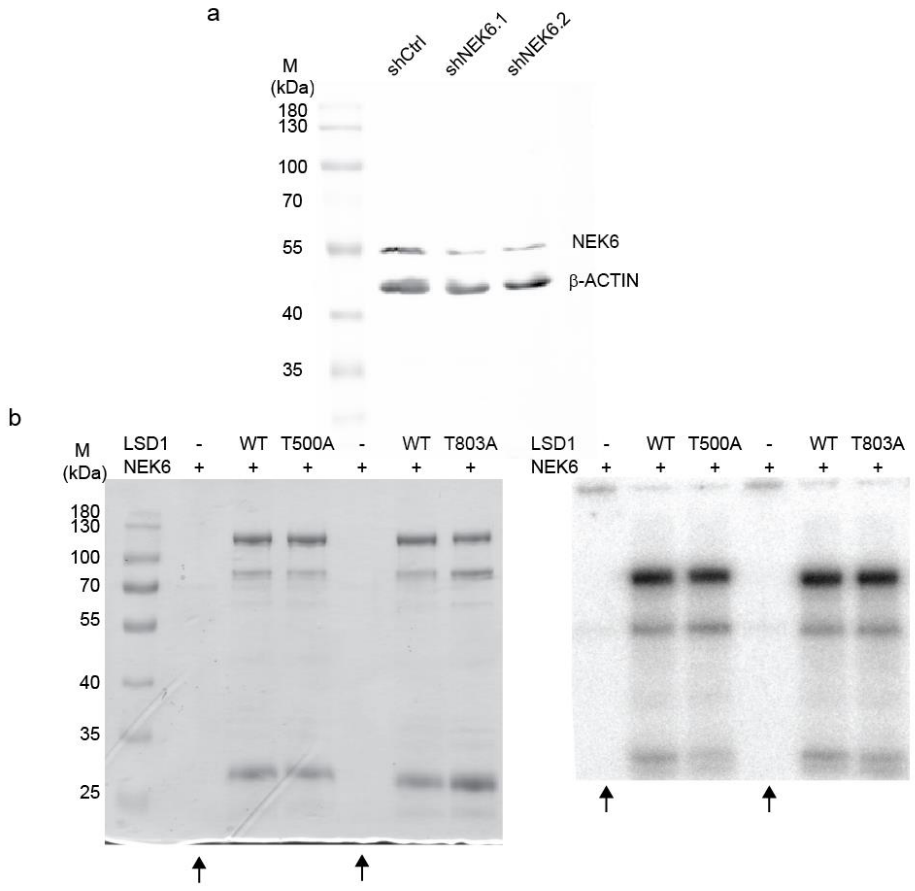

Supplementary Fig. 8: a) Uncropped Western Blot picture related to Supp. Fig 1a. b) Uncropped SDS-PAGE picture and corresponding radiography picture related to Supp. Fig. 2e. Arrows mark lanes that are not shown in the connected figures.
