# Supplementary material for: The kinase NEK6 positively regulates LSD1 activity and accumulation in local chromatin sub-compartments
Source: Commun Biol. 2024 Nov 10;7:1483. doi: 10.1038/s42003-024-07199-x (PMC11551153; doi:10.1038/s42003-024-07199-x)
Supplement: Supplementary file 3 — Description of Additional Supplementary File [file 42003_2024_7199_MOESM3_ESM.pdf]

Description of additional supplementary files

File name: Supplementary table 1:

Description: Detection matrix of the LC-MS/MS analysis. 94% of all amino acids of LSD1 had been detected after protein digestion with Glu-C, trypsin and ProAnalase including all potential phosphorylation target sites (S, Y, T) except T783 and S787.

File name: Supplementary video 1, 2 and 3

Description: (connected to Fig. 3d) Coalescence of IDR droplets. Three bright field LSD1 IDR-mV in vitro coalescence events.

File name: Supplementary video 4 and 5

Description: (connected to Fig. 5c) Corelet droplet formation of LSD1 and NEK6. Representative videos of cellular phase separation of LSD1 (video 3) and NEK6 (video 4) in HEK293 cells. Interval of 0.25 s over 20s of blue light activation.

File name: Supplementary video 6 and 7

Description: (connected to Fig. 5d) Intracellular coalescence events of NEK6 Corelet droplets. Representative videos of NEK6 Corelet coalescence events in in HEK293 cells. Interval of 0.25 s over 20s of blue light activation.

File name: Supplementary video 8 and 9

Description: Corelet droplet dissolution upon blue light removal. Representative videos of LSD1 Corelet disassembly in HEK293 cells upon removal of blue light. Blue light was added for 20 s and dissolution was imaged over 2 min in 5 s interval.

File name: Supplementary Data

Description: Excel sheet containing all numerical source data behind the graphs in the main and supplementary figures of this paper sorted into separate tabs for each sub figure.
